# Effects of Head-Down Bed Rest on the Executive Functions and Emotional Response

**DOI:** 10.1371/journal.pone.0052160

**Published:** 2012-12-17

**Authors:** Qing Liu, Renlai Zhou, Shanguang Chen, Cheng Tan

**Affiliations:** 1 Beijing Key Lab of Applied Experimental Psychology, School of Psychology, Beijing Normal University, Beijing, China; 2 State Key Laboratory of Cognitive Neuroscience and Learning, Beijing Normal University, Beijing, China; 3 China Astronaut Research and Training Center, Beijing, China; Université de Montréal, Canada

## Abstract

Prolonged bed rest may cause changes in the autonomic nervous system that are related to cognition and emotion. This study adopted an emotional flanker task to evaluate the effect of 45 days -6° head-down bed rest (HDBR) on executive functioning in 16 healthy young men at each of six time points: the second-to-last day before the bed rest period, the eleventh, twentieth, thirty-second and fortieth day during the bed rest period, and the eighth day after the bed rest period. In addition, self-report inventories (Beck Anxiety Inventory, BAI; Beck Depression Inventory, BDI; Positive Affect and Negative Affect Scale, PANAS) were conducted to record emotional changes, and the participants’ galvanic skin response (GSR), heart rate (HR) and heart rate variability (HRV) were assessed as measures of physiological activity. The results showed that the participants’ reaction time on the flanker task increased significantly relative to their responses on the second-to-last day before the period of bed rest, their galvanic skin response weakened and their degrees of positive affect declined during the bed rest period. Our results provide some evidence for a detrimental effect of prolonged bed rest on executive functioning and positive affect. Whether this stems from a lack of aerobic physical activity and/or the effect of HDBR itself remains to be determined.

## Introduction

Head-down bed rest (HDBR) has proven to be a reliable means of simulating the weightlessness that occurs during space flight and replicating its effects. Because continuous bed rest is a form of sensory deprivation, it has been used as a model for examining the functional impairments and later recovery that result from prolonged exposure to the headward fluid shift and other aspects of weightlessness [1]. Accordingly, both the European Space Agency (ESA 2004) and the National Aeronautics and Space Administration (NASA 2008) regard HDBR as an important method of testing space flight effects in their in-progress plans to send humans to Mars, and it is in this context that the ESA has recently initiated a 5-year bed rest study program (ESA 2006) [2]. The results from experiments conducted during the past few years and in which HDBR were used to simulate weightlessness have presented an overview of problems with physiological adaptability and changes in performance that occur during HDBR. However, researchers have paid relatively little attention to psychological factors even though an understanding of the psychological effects of weightlessness may be closely related to both ensuring the safety of the astronauts and accomplishing the missions of the flights [3], [4].

Long-duration space flights, the construction of the International Space Station (ISS) and crewmember factors have already indicated that exposure to microgravity may impair normal psychological functions, including cognitive and emotional functions, in individuals [5], [6]. Accordingly, many researchers have investigated psychological aspects in bed rest experiments, but the outcomes of these experiments lacked consistency [2]. Some reports indicated bed rests did a detrimental effect on cognitive functioning. For example, a study of Lipnicki, Gunga, Belavý and Felsenberg found that prolonged bed rest had a detrimental effect on executive function. Their results showed that participants’ tendencies to engage in high-risk activities increased and reaction time latencies were longer under HDBR [7]. Other studies have suggested that prolonged bed rest had no effect on cognitive performance. For instance, Ishizaki, Fukuoka and Tanaka examined the effect of simulated microgravity that resulted from exposure to a prolonged period of 6° HDBR on executive functions in 12 healthy young men. They found no significant differences between the baseline and the test results on day 16 for any of the tests that they conducted [8]. Certainly, some studies found an improvement in cognitive performance under bed rest. For example, Traon et al. discovered that participation in 28-day HDBR failed to result in worsened cognitive performance. They observed that participants’ performance on tests of short-duration memory and automated perceptual attention were enhanced [9].

Studies of the influence of exposure to prolonged bed rest on individual emotions have also failed to reach a consensus. Ishizaki et al. showed levels of depressive and neurotic symptoms were enhanced after 20-day HDBR [10]. Qin et al. reported that the 21 healthy young males who participated in their 60-day HDBR exhibited a tendency toward having high-low-high-low cycles of negative emotions during the bed rest period [11]. By contrast, a recent study in which participants underwent 15-day HDBR did not show any symptoms of clinical depression or anxiety during bed rest, and the positive and negative emotions of the participants were within normal range [12]. Thus, consensus on the effects of HDBR on cognition and emotion has not been reached.

There are many potential causes to which the inconsistent results of the aforementioned studies could be attributed. First, because HDBR simulated an extreme microgravity environment, the external validity of the studies and the possibility for duplicating these results were both low [2]. In addition, task difficulty was an important operating variable in the measurement of cognitive function. Tasks which were likely to generate practice effects could account for improvement of performance in some studies, so reports of detrimental cognitive effects may have been more common for tasks that need automatic processing [13]. Moreover, variation in the durations of the experiments exerted different impacts on individual rhythms and cognitive functioning. For example, short duration space flights that only lasted one to two weeks generally did not affect the astronauts’ cognitive performance (especially advanced cognitive functions) and psychological capacities [14]. Most astronauts were able to endure space-related stressors in the short duration space flights. However, long duration space flights that lasted for six or more weeks exerted a more complicated influence on the psychological and cognitive performances of the astronauts [15]. Because in long duration space flights, individuals’ adaptive abilities were exhausted, and they were exposed to new physiological and psychological stress factors.

Some conclusions can be drawn on the basis of the above mentioned studies. First, studies of short or long duration space flights (bed rest) commonly concerned the variation of individual executive functioning and emotion. Colcombe and Krame thought this generality could be interpreted for executive functioning that were particularly affected by changes in physical activity and fitness levels and which turned out to be the most likely cognitive ability affected by bed rest [16]. Thayer, Hansen, Evelyn and Bjorn found that studies about the relation of cognition and prefrontal neural function often were investigated in negative affect situations or together with negative affect such as depression and anxiety [17]. Therefore, in our study we tested individual executive functioning and emotion simultaneously under prolonged bed rest. We adopted an emotional flanker task to evaluate variations of individuals’ executive functioning abilities in response to emotional stimuli and to elucidate the relation between individuals’ executive functioning and emotion under prolonged bed rest. Secondly, the correlation between individual vagus nerve activity (which could be reflected in the high frequency (HF), vagal component of HRV [18]) and prefrontal cognitive function [19], together with the consistency between vagus nerve activity levels and emotional changes [20] that were reported in previous studies both indicated that the vagus nerve activity was closely connected with both executive functioning and emotion. HDBR had effects on cardiac vagal tone was commonly known [3]. And the GSR was a measure of the electrical resistance of the skin which was proportional to sweat secretion and acted as an indicator for sympathetic activation due to the stress reaction [21]. So the measurements of automatic nervous system (ANS) which were performed by GSR and HRV could be used to represent the relation of executive functioning and emotion under HDBR.

Above all, we examined trends in the variations of individual executive functioning and emotion during 45-day -6° HDBR. Specifically, we administered an emotional flanker task before, during and after the period of HDBR to assess the variation of executive functioning. The participants’ GSR, HR and HRV were measured as indicators of physiological changes simultaneously. Moreover, participants were given self-report inventories to record their emotional changes at each test point as well. Both subjective and objective measurements were combined to investigate the relation of individual executive functioning and emotion under prolonged HDBR. Our theoretical rationale led us to hypothesize that prolonged HDBR may have a detrimental effect on individual executive functioning and emotion, and HRV may be an effective index to reflect individual executive functioning and emotion changes.

## Methods

### Ethics

All participants provided written informed consent to participate in the present experiment. Experimental procedures were approved by the Institutional Review Board of the State Key Laboratory of Cognitive Neurosciences and Learning of Beijing Normal University.

### Participants

Sixteen healthy non-smoking men provided written informed consent to participate in the present experiment. However, data from one of the participants were not recorded due to a malfunction of the multipurpose polygraph, so the valid data were collected from a total of 15 volunteers. The 15 healthy men had a mean age of 26.33 years old (*SD = *4.13, range: 20–34 years), with no history of any chronic or recent acute illness and had normal vision. The mean body mass of the participants was 62.20 kg (*SD = *5.56, range: 55–75 kg), and their mean height was 170.13 cm (*SD* = 4.09, range: 160–175 cm). The participants were right-handed, non-athletes and none of them were allowed to use medication, tobacco, or caffeine-containing drinks during the experiment.

### Procedure

The current bed rest experiment lasted for a total of 65–70 days, which comprised a pre-HDBR period of 10 days, a -6° HDBR period of 45 days to simulate prolonged exposure to weightlessness and a post-HDBR recovery period of 10–15 days. The duration of the post-HDBR period depended on the recovery time of each volunteer. During the 45 days bed rest period, participants were restricted to absolute head-down recumbence even while eating, excreting, bathing and sleeping without pillows. They were freely permitted to watch television and videos, to listen to CDs or the radio, to play games, and to read books and magazines. Six to eight participants were housed together in each of the rooms in which the experiment was conducted, the beds were separated by moveable curtains, and the room temperature was kept at a comfortable temperature of approximately 25°C. During the pre-HDBR period, each participant received a balanced diet for 10 days to maintain a stable nutritional state. To avoid the influence of nutrition on the results of the experiment, the participants were fed a fixed number of calories per kg of lean body mass during the HDBR and post-HDBR, but their daily water intake was not limited. During the HDBR period, physicians monitored the physical conditions of the participants, including taking measurements of the blood pressure, heart rate, breathing rate, body temperature, water consumption, urinary output and general health condition of each participant. The daily schedule was as follows: awakening at 06∶00 am, breakfast at 06∶30–07∶00 am, lunch at 11∶30–12∶30 am, afternoon nap at 12∶30–13∶30 pm and dinner at 17∶30–18∶30 pm. At 22∶00 pm, the lights were turned off to maintain the participants’ day-night rhythms. The rest time of the day was for experiments and exercise. Four kinds of tests (the emotional flanker task; the Beck Depression Inventory, BDI; the Beck Anxiety Inventory, BAI; and the Positive Affect and Negative Affect Scale, PANAS) were performed at each of six time points: the second day of the pre-HDBR, the eleventh (HDBR11), twentieth day (HDBR20), thirty-second day (HDBR32) and fortieth days (HDBR40) of the bed rest period, and the eighth day of the post-HDBR, at 14∶00–18∶00 pm. Exercise included bicycle power meter exercise and lower body negative pressure (LBNP). To be consistent with tests that were conducted during the HDBR period, the tests of pre-HDBR and post-HDBR were conducted while participants were lying in a horizontal position. The participants completed a Post-survey about their bad feelings towards the HDBR on the post-HDBR.

### Materials

#### Emotional flanker task

**Figure 1:**
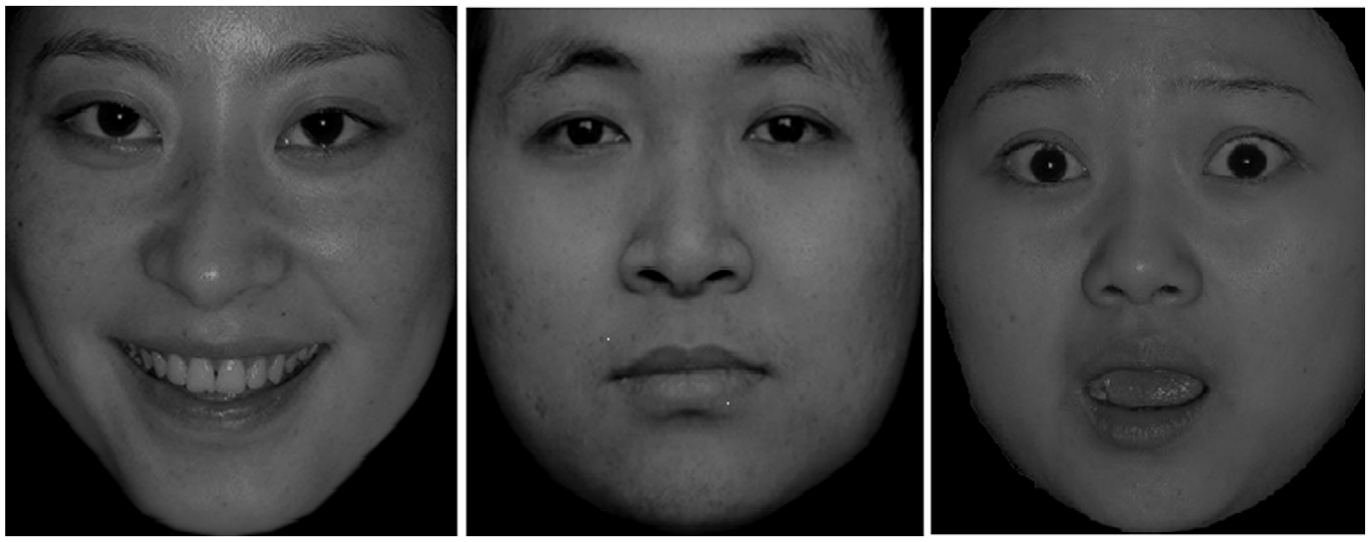
Emotional picture samples (happy, neutral and fear).

The E-Prime software program was used to run the emotional flanker task, and we collected physiological data from the participants with a MP150 multipurpose polygraph five minutes prior to the start of the emotional flanker task. All of the stimulus pictures were monochromatic and had black backgrounds. All of the emotional facial pictures were taken from the Chinese Facial Affective Pictures System [22]; an example is shown in Figure 1. Three categories of facial pictures were used – happy (positive), neutral, and fear (negative), and 20 pictures (10 male faces and 10 female faces) from each category were included in the stimulus set. The stimulus picture for each trial consisted of three emotional faces – one face was used as a target, and two faces were used as distractors; a total of 185 trials were presented. The procedure consisted of one practice block and two experimental blocks. Seventeen of the 185 trials were randomly selected for inclusion in the practice block, and the remaining 168 trials were divided into two blocks, each of which was comprised of 84 trials. Participants were told three pictures would appear on the screen and they should pay attention to the center picture regardless of the other two pictures. The following further instructions were given: if the center picture was a happy face, press the left button, if the center picture was a neutral face, press the middle button, if the center picture was a fear face, press the right button. The participants had to quickly press in order to ensure the correct premise. Each subsequent trial began immediately after the participant responded to the current trial. Each of the stimulus pictures could also be grouped into one of the two conditions: a congruent condition and an incongruent condition. In the congruent condition, the emotion of the center picture was congruent with the other two pictures. For example, in a trial, if the center picture was a happy face and the other two pictures were also happy faces, the trail was the congruent condition. By contrast, in the incongruent condition, the center picture was a happy face and the other two pictures were not happy faces. Each trial was initiated by the presentation of a small cross (+) in the center of the screen for a total of 500–800 ms, followed by a 500-ms presentation of a stimulus picture and an 800- to 1200-ms white response screen. The relevant response keys were counterbalanced between trials, and participants’ responses were recorded via a response box. Prior to statistical processing, the raw data were filtered, and incorrect responses and outliers (i.e., data that fell more than three standard deviations away from the mean) were deleted. The trails were randomly set in each block. As described by Lipnicki, Gunga, Belavý and Felsenberg [7], we used the same procedure across six test time points to assess possible variation of individual executive functioning under HDBR.

#### Beck anxiety Inventory and beck depression inventory

The Beck Anxiety Inventory (BAI) and the Beck Depression Inventory (BDI) have been used to measure anxiety and depression among research participants, and the validity and reliability of the Chinese version are well established [23]. The BAI is comprised of 21 questions that assess the degree to which a participant experiences each symptom of anxiety. The BAI uses a four-level scale in which one means no discomfort, two means mild discomfort that is of little annoyance, three indicates a moderate but endurable level of discomfort, and four indicates a level of discomfort that is severe and almost or completely unbearable. The BDI has 21 items, each of which presents a category. The description of each category divides into four levels and scores zero to three for the different levels. We used standardized scores for further analyses of BAI; we calculated the total raw scores for the 21 items and then used the equation Y = int (1.19X) [24] to transform them into a standardized score. In general, a total BAI score of ≥45 was considered indicative of clinical anxiety. However, we used raw scores for further analyses of BDI, the range was 0–69, with higher scores indicating more serious depression. Specifically, total raw scores between 0–19 were considered to be in the mild range, between 20–29 moderate and 30–69 were considered major depression.

#### Positive affect and negative affect scale

The Positive Affect and Negative Affect Scale (PANAS) is comprised of 20 items and includes two emotional dimensions: positive and negative. The Chinese version of the PANAS has well-established validity and reliability [25]. Ten items were used to evaluate negative affect and the remaining 10 items were used to evaluate positive affect. The raw score for each item ranged from one to five.

### Data Collection

Physiological activity data were collected via MP150 system amplifier modules that included specific modules for the acquisition, conversion, amplification and storage of signals. We used the digital filter to process the physiology signals. Specifically, we chose the spectrum analysis to extract the wave band of interest. The bipolar electrodes that were used to collect the galvanic skin response (GSR) data were attached to the index and middle fingers of the participants’ left hands (VIN+ and VIN–, respectively). The amplifier gain of the GSR was 5µmho/V, the high-pass filter was DC and the low-pass filter was 1 Hz. The sample rate was 250 Hz and the units were in µmho. The HR of each participant was obtained on the basis of the R-R interval that was immediately extracted from the electrocardiograph (ECG) signal. The ground (GND) was connected to the right lower limb, the VIN+ was connected to the left lower limb and the VIN–, which showed the electrode connections to the ECG for the lead measurements, was connected to the left upper limb. The amplifier gain was 500, the high-pass filter was 0.5 Hz, the low-pass filter was 35 Hz and the sample rate was 500 Hz. The ECG units were beats per minute (bpm). The resting-state GSR and ECG data were recorded while the participants were in comfortable and relaxed states, and recorded serially for 5 minutes after all of the components of the apparatuses had been attached.

### Statistical Analysis

Statistical analyses were performed using version 16.0 of the SPSS software program. Statistically significant differences were assessed using one-way repeated-measures ANOVAs, and a p-value of *p*<0.05 was considered significant. Strength of the effect was assessed by eta-squared (*η2*) and the correction was done by Greenhouse-Geisser coefficient. The AcqKnowledge 4.1 software program was used to extract and analyze the GSR, HR and HRV data. ECG data were collected at 5-minute intervals. From these data, the HRV spectral information which included the high frequency (HF) component (0.15–0.40 Hz), the low frequency (LF) component (0.04–0.15 Hz) and the balance of the frequencies (LF/HF), was obtained via Fast Fourier Transforms (FFTs) of the ECG signals.

## Results

### Changes in Individual Executive Functions During Conditions of -6°HDBR

The participants’ mean reaction time (RT) and accuracy (ACC) in performing the emotional flanker task are presented in Table 1. We used a repeated-measure ANOVA in which the test time points (the second day of the pre-HDBR; the eleventh day, HDBR11; the twentieth day, HDBR20; the thirty-second day, HDBR32; the fortieth day of the bed rest period, HDBR40; the eighth day of the post-HDBR) were the independent variables and the participants’ mean RT and ACC for each time point were the dependent variables. The ANOVA showed no significant differences in mean task performance between six time points in terms of ACC (*F*_(5, 70)_ = 0.428, *p* = 0.828, *η2* = 0.030); however, the mean RT for the emotional flanker task on each of the six testing time points were significantly different (*F*_(5, 70)_ = 5.084, *p*<0.001, *η2* = 0.266), and the quadratic increase was also significant (*F*_(1, 14)_ = 19.793, *p* = 0.001, *η2* = 0.586). Further analysis revealed that the mean RT of the pre-HDBR was significantly shorter than HDBR11, HDBR20, HDBR32 and HDBR40. In addition, the mean RT of post-HDBR was shorter than the mean RT of HDBR40, but not significant. There was no significant difference in mean RT between pre-HDBR and post-HDBR. Moreover, pre-HDBR, the mean RTs for the congruent and incongruent conditions of emotional flanker task showed significant differences, *F* = 24.17, *p*<0.001; HDBR11, the differences between ACC(*F* = 5.57, *p* = 0.033)and RT (*F* = 31.60, *p*<0.001) in the congruent and incongruent conditions were significant, which indicated participants’ ACC and RT of the congruent condition were significantly higher and shorter respectively than in the incongruent conditions.

**Table 1:** Flanker task performance (accuracy and reaction time) of the participants at each of six time points.

	Time Points					
Executive function	pre-HDBR	HDBR11	HDBR20	HDBR32	HDBR40	post-HDBR
	M SD	M SD	M SD	M SD	M SD	M SD
Accuracy	0.72(0.22)	0.73(0.14)	0.70(0.13)	0.76(0.11)	0.71(0.16)	0.70(0.20)
Reaction time	517.90(80.82)	584.09(64.37)	602.69(40.01)	600.52(47.96)	583.92(68.73)	555.28(115.84)

### Changes in the Emotional Self-report Inventory Scores of the Participants Under Conditions of -6°HDBR

#### Changes in the anxiety and depression scores of the participants at different test time points

**Figure 2:**
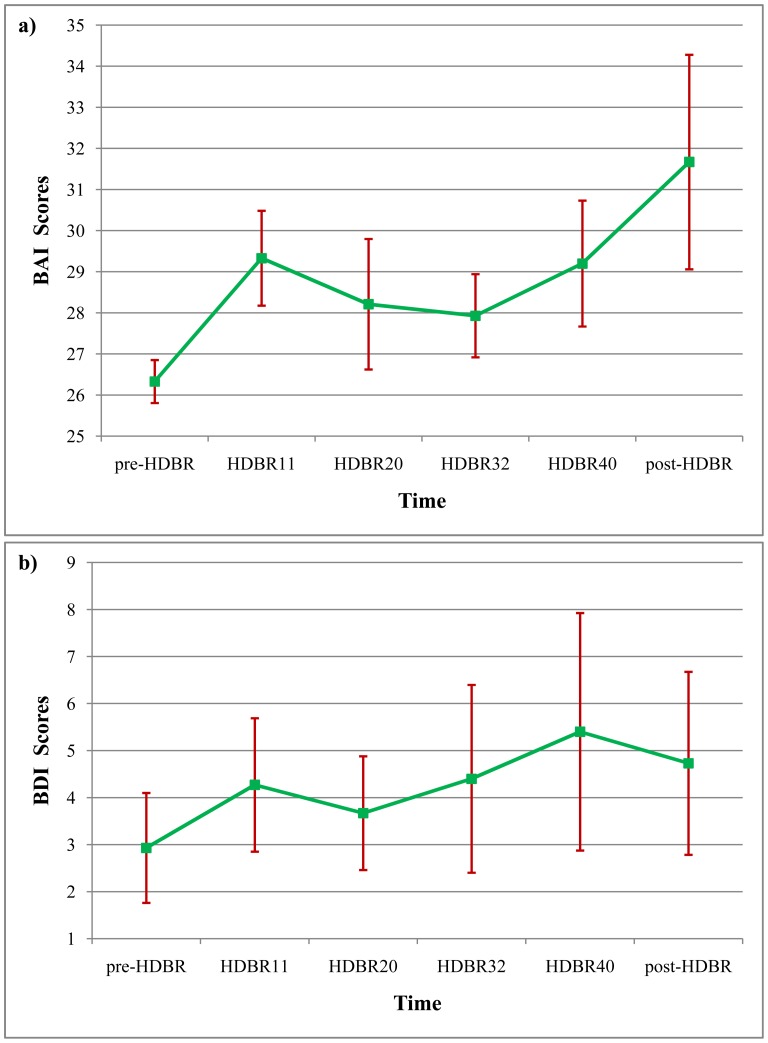
Participants’ Beck Anxiety Inventory (BAI) and Beck Depression Inventory (BDI) scores at different time points. a) Participants’ performance on BAI at six time points (pre-HDBR, HDBR11, HDBR20, HDBR32, HDBR40, post-HDBR). b) Participants’ performance on BDI at six time points.

We analyzed depression and anxiety scores of participants at different times throughout the duration of the experiment, and we found anxiety scores ranged from 24 to 30 on the pre-HDBR, 24 to 41 on the HDBR11, 24 to 44 on the HDBR20, 24 to 36 on the HDBR32, 24 to 44 on the HDBR40, and 24 to 61 on the post-HDBR. These scores are shown in Fig. 2 (a). Similarly, the BDI scores of the participants ranged from zero to 15 on the pre-HDBR, zero to 21 on the HDBR11, zero to 15 on the HDBR20, zero to 26 on the d HDBR32, zero to 34 on the HDBR40, and zero to 22 on the post-HDBR. These data are depicted in Fig. 2 (b). We used a repeated-measure ANOVA to investigate the statistical significance of differences in anxiety and depression scores, and the results showed no significant differences in the mean anxiety scores at the different time points (*F*_(5, 70)_ = 1.586, *p* = 0.176, *η2* = 0.109). However, the cubic increase was significant (*F*_(1, 14)_ = 5.406, *p* = 0.038, *η2* = 0.294). Similarly, the main effect of the mean depression scores that were recorded at different time points was not significant (*F*_(5, 70)_ = 0.495, *p* = 0.779, *η2* = 0.034). The results showed that 45-day HDBR didn’t evoke clinical levels of anxiety and depression.

#### Changes in the positive affect and negative affect scores (PANAS) of the participants at different test times

**Figure 3:**
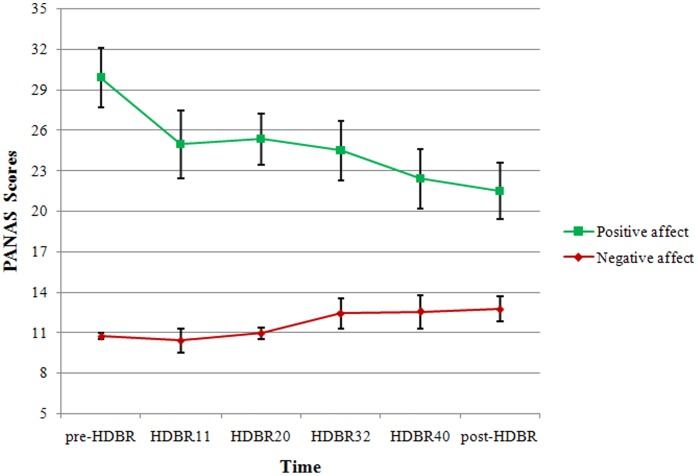
Participants’ positive affect and negative affect scores at different time points.

We used a repeated-measure ANOVA to analyze the participants’ positive affect and negative affect scores at different time points. Results indicated that the main effect of positive affect at different time points was significant (*F*_(5, 70)_ = 2.788, *p* = 0.024, *η2* = 0.166) and a linear increase was also significant (*F*_(1, 14)_ = 7.968, *p* = 0.014, *η2* = 0.363). Further analysis showed that the individual positive affect scores declined as soon as the HDBR started (the value of HDBR11), and then rose on HDBR20, but declined again on HDBR32, positive affect scores continued to decline and on HDBR40 were significantly lower than the pre-HDBR (*p*<0.05). Compared with the HDBR40, the participants’ positive affect scores declined on post-HDBR, and the scores on post-HDBR were also significantly lower than pre-HDBR (*p*<0.05). The fluctuation of participants’ positive affect showed a wave of high-low-high-low across the HDBR (before, during and after), which specifically corresponded to the time points to pre-HDBR, HDBR11, HDBR32 and post-HDBR. In contrast, there were no significant differences in the negative affect scores at different time points (*F*_(5, 70)_ = 2.220, *p* = 0.062, *η2* = 0.137). These data are presented in Fig. 3.

### Changes in the Participants’ Physiology under Conditions of -6°HDBR

#### Changes in the participants’ resting-state galvanic skin responses at different test time points

**Figure 4:**
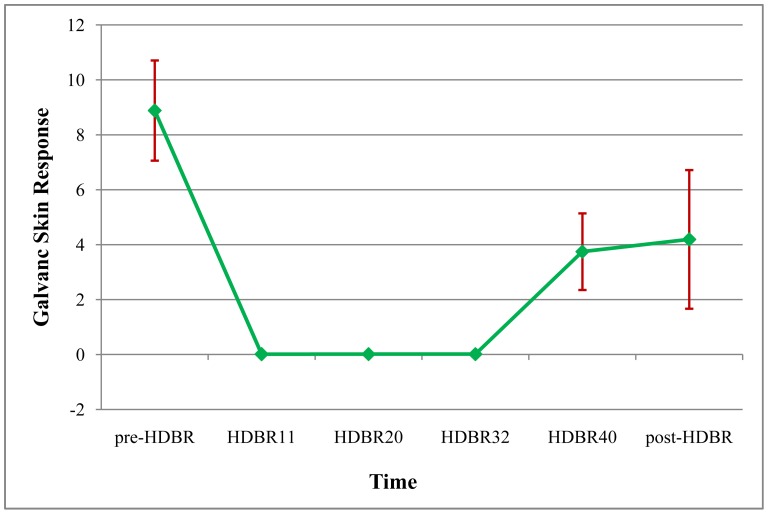
Galvanic skin response of the participants at different time points. Notes: The mean values of different time points (pre-HDBR, HDBR11, HDBR20, HDBR32, HDBR40, and post-HDBR) were 8.89, 0.012, 0.015, 0.015, 3.75 and 4.19µmho.

Repeated-measure ANOVA was used to analyze differences in the GSR of the participants at different test time points (pre-HDBR, HDBR11, HDBR20, HDBR32, HDBR40, post-HDBR); we found a significant main effect of the test time points (*F*_(5, 70)_ = 6.573, *p*<0.001, *η2* = 0.319). We also found a significant quadratic increase (*F*_(1, 14)_ = 11.927, *p* = 0.004, *η2* = 0.460), the variation of GSR are shown in Fig. 4. Further analysis indicated GSR of participants significantly declined (*p*<0.05) during the HDBR period (HDBR11, HDBR20, HDBR32, HDBR40) relative to GSR obtained on the pre-HDBR. In addition, the GSR on the HDBR40 significantly increased (*p*<0.05) in comparison to the GSR that was obtained on the HDBR11, HDBR20 and HDBR32, and the GSR on the post-HDBR increased compared to HDBR40. However, the GSR on the post-HDBR was not significantly different from the GSR on pre-HDBR, the HDBR40 or any time point during the HDBR.

#### Changes in the resting heart rate and heart rate variability of the participants at different test time points

The resting HR and HRV of the participants at different test time points are presented in Table 2. Repeated-measure ANOVAs were used to analysis participants’ HR and HRV across six time points (pre-HDBR, HDBR11, HDBR20, HDBR32, HDBR40; post-HDBR). The results showed that neither the main effect of HR nor different components of HRV were significantly different across the six time points. Although the main effect of HF (*F*_(5, 70)_ = 1.309, *p* = 0.270, *η2* = 0.086) and LF (*F*_(5, 70)_ = 1.233, *p* = 0.303, *η2* = 0.081) were not significant, there were both a significantly linear increase for HF (*F*_(1, 14)_ = 6.462, *p* = 0.023, *η2* = 0.316) and LF (*F*_(1, 14)_ = 7.037, *p* = 0.019, *η2* = 0.335). Therefore, the linear trends of HF and LF may provide some evidence for HRV variation across different time points under prolonged bed rest.

**Table 2:** Changes in the resting heart rate and the heart rate variability of the participants at different test times.

	Time Points					
	pre-HDBR	HDBR11	HDBR20	HDBR32	HDBR40	post-HDBR
	M SD	M SD	M SD	M SD	M SD	M SD
Heart rate	76.77(28.60)	65.16(8.98)	68.13(8.46)	69.80(8.53)	73.43(8.08)	76.06(11.66)
Low frequency	5.96(2.80)	5.74(3.80)	6.22(4.88)	4.55(1.99)	5.01(3.02)	3.90(2.31)
High frequency	1.50(0.63)	2.35(2.66)	2.38(3.74)	1.16(0.53)	1.43(1.15)	0.98(0.58)
LF/HF	3.76(0.87)	3.61(0.63)	3.58(0.91)	4.02(0.75)	3.92(0.88)	3.91(0.67)

Notes: high-frequency and low-frequency data that are shown in table 2 were magnified 1000 times relative to the raw data so that they could be observed clearly.

## Discussion

The results indicated that the participants’ RT in the flanker task increased significantly, the GSR and positive affect scores decreased significantly during the HDBR compared to the pre-HDBR. Post-HDBR, the positive affect scores decreased significantly compared to pre-HDBR, but the RT and GSR did not differ significantly between the pre-HDBR and post-HDBR. Meanwhile, there were no significant differences in the mean flanker task ACC, HR, HRV, depression scores, anxiety scores and negative affect scores obtained at different time points (before, during and after HDBR). These results are consistent with the assumption that the cognitive abilities of participants, specifically their executive functioning abilities, would be impaired as a result of long-duration HDBR, meanwhile their emotion and physiological activity would also be influenced. HRV was identified as a sensitive physiological index which reflected the relation of changes in executive functioning and emotion under prolonged bed rest. The findings may be due to HDBR itself. HDBR decreases physical activity and bioactivity, slows down the circadian rhythms, finally increases reaction time [16]. Therefore, we speculate that the increase of the flanker task RT during the HDBR may result from physiological changes caused by the prolonged HDBR. The delay of reaction time may be considered as the detrimental effect of prolonged HDBR on executive functioning.

Our results are inconsistent with the findings of a study by Lipnicki et al. who found that, participants’ RT on a flanker task were not significantly longer during HDBR when compared with the RT prior to the start of 60-day HDBR [8]. The discrepancy between our experiment and the study of Lipnicki et al. might be attributed to the difference of the stimuli type used in the two experiments. Lipnicki adopted a version of the classic flanker task which used arrows as stimulus pictures (a picture formed by three arrows, each of which directed to different orientations) and in which the participants were required to respond to the orientation of the middle arrow as quickly as possible. According to the study by Svensson and Wilson, tasks like the classic flanker task which involve automatic processing, are likely to generate a practice effect [13]. Therefore, improvement on the flanker task ACC in Lipnicki’s study may be due to practice rather than the effect of HDBR. The discrepancy also reflected that when talking about effect of HDBR on executive functioning, task difficulty should be taken into consideration. Based on the perspectives of Svensson and with the intent of reducing practice effect, we used a modified flanker task (the emotional face flanker task). An emotional dimension (including happy, neutral and fear variants) that increased task difficulty was added to test participants’ executive functioning of emotion stimuli under prolonged HDBR, which produced a detrimental result. Therefore, the effect of prolonged HDBR on individual executive functioning might be detrimental. The discrepancy in the results of executive functioning between our study and a 20-day bed rest research performed by Ishizaki might account for the duration difference of HDBR. Ishizaki indicated that individual executive functioning didn't have significant changes between the baseline and day 16 of HDBR [10]. This difference was also observed on individual emotion under HDBR.

The current study showed that the positive affect scores (PANAS) of the participants varied across the pre-HDBR, HDBR and post-HDBR periods. This result of emotion was consistent with the study of Qin [11]. Qin posited that long-duration exposure to HDBR would influence individual emotional state which would perform a wave of high-low-high-low variation on negative affect; similarly our result of positive affect turned out to be a low-high-low-high trend which illustrated that long-duration HDBR had an effect on individual emotion regardless of positive affect or negative effect. Meanwhile, on the basis of using the same materials for emotion test, our study was inconsistent with Chen [12]. Although anxiety, depression and negative affect did not change, positive effect under prolonged HDBR across six different test points changed significantly in our study, while Chen found that no emotion changed (anxiety, depression, positive and negative affect) under 15-day HDBR across different test points (five days before HDBR, HDBR5, HDBR10, five days after HDBR). These differences in the result could be attributed to the difference of duration which further demonstrated that failing to evoke participants’ clinical anxiety and depression under HDBR (45-day and 15-day), prolonged HDBR may have a detrimental effect on participants’ positive affect that then decreased following the process of HDBR.

In the present study, we took various physiological measurements (GSR, HR and HRV) in order to reflect individual automatic nervous system (ANS) variation under prolonged HDBR across six different time points. First, there was a fluctuation of GSR across our experiment compared to pre-HDBR; participants’ GSR weakened as soon as they entered the HDBR and lasted to HDBR32. The GSR then strengthened and significantly strengthened on the 40d of HDBR. After the HDBR, participants’ GSR continued to strengthen but not significantly in comparison with HDBR40. Perhaps we can use the value of Leon et al. to explain the fluctuation of GSR. Leon thought under extreme environments like outer space, individuals would perform a “third-quarter phenomenon”, which definitely described as anxiety during a prophase, boredom during a metaphase and terminal excitation [4]. The variation of GSR in our study was in accordance with the third-quarter phenomenon, which may reflect an individual’s adaptation process under prolonged HDBR.

In the present study, the GSR variation was also consistent with the flanker task variation, which could be interpreted that prolonged HDBR may have an effect on individual physiology activities like GSR and that individual physiology and executive functioning are connected to each other. This connect could be explained by the conclusions of Hochey who thought that individual performance, especially higher cognition in outer space, would be influenced by the arousal and variation pattern of physiology [26]. Secondly, the consistency between HRV and flanker task ACC in our study confirmed Thayer’s speculation regarding the relation between vagus nerve activity and executive functioning. Thayer and co-workers thought that the resting HRV, which reflected an individual’s vagus nerve activity, was related to his/her performance on an executive control task [17]. Similarly, we found evidence for a similar trend in the participants’ positive affect scores and HRV under pre-HDBR and post-HDBR that can be explained by a study of Richard. Richard held a view that the area in which activity was evoked by emotional stimuli (the prefrontal cortex) was also the neural basis of the ANS [18]. Because the HRV (the HF) reflected the activity of the vagus nerve in the ANS, the consistency between the HRV and positive affect scores in the present study suggests that there may be a connection between the participants’ emotion and their HRV. Thus, under prolonged HDBR, an individual’s HRV may turn out to be an effective ANS index that is related to the cognition and emotion. Based on these findings of HRV, maybe when selecting or training people for future space flights, HRV could be an extra index to show some evidence of one’s ability to perform (cognition, emotion and ANS performance) in extreme environments like the outer space.

Overall, prolonged HDBR had a detrimental effect on individual executive functioning, emotion and physiological activity were obtained under the HDBR condition an analog of space. Further studies should pay more attention to the real space flight and then combine the results of HDBR to fully demonstrate the effect of space on individual.
